# Unsupervised Frailty Intervention by Digitalized Exercise Coaching: A Feasibility Study

**DOI:** 10.3390/s25123674

**Published:** 2025-06-12

**Authors:** Changseok Lee, Jehun Lee, Heeyoung Jeong, Haeram Lee, Eunah Wang, Gyungyoon Baek, Hyeri Shin, Seongjun Yoon

**Affiliations:** 1 DYPHI Research Institute, DYPHI Inc., Daejeon 34134, Republic of Korea; ckdckd145@dyphi.com (C.L.); jehuning@dyphi.com (J.L.); heeyoung@dyphi.com (H.J.); nooroongy@dyphi.com (H.L.); eunah@dyphi.com (E.W.); gybaek@dyphi.com (G.B.); 2 Department of Gerontology, Kyung Hee University, Yongin-si 17104, Republic of Korea; zisoa@khu.ac.kr

**Keywords:** frailty, unsupervised intervention, digital intervention, physical performance, SPPB

## Abstract

Multi-component interventions have been demonstrated to be an effective method for the prevention of frailty. Nevertheless, they have not yet been widely adopted in practice due to considerable resource and labor demands associated with their administration. To overcome the limitations, this study aimed to determine the feasibility of unsupervised intervention based on digital therapy. A mobile application has been developed to deliver multi-component exercise coaching to frail older adults. A total of 30 participants (with a mean age of 72.10 ± 4.54 years) were recruited from two community centers and used the mobile application for 12 weeks without supervision. Prior to utilizing the mobile application, each participant received an initial education. Outcomes of the Short Physical Performance Battery (SPPB), the Korean version of the Fatigue, Resistance, Ambulation, Illnesses, and Loss of weight (K-FRAIL) scale, activities of daily living (ADL), instrumental activities of daily living (IADL), self-efficacy, and depression, were evaluated before and after using the mobile application. Significant improvements in the mean SPPB score (9.6 ± 1.7 to 11.7 ± 0.5) and depression (3.23 ± 3.08 to 2.00 ± 2.11) were observed. The total adherence rate of all participants was 86.1%. No statistically significant differences were observed in the remaining outcomes. These findings suggest that the unsupervised intervention could be a viable option for older adults.

## 1. Introduction

Frailty is a geriatric syndrome associated with aging that increases the risk of falls, hospitalization, disability, mortality, and loss of independence in older adults [1]. Multi-component interventions, including exercise, nutrition, and social engagement programs, have been shown to be effective in preventing and managing frailty [2,3,4,5]. However, implementing multi-component interventions on a large scale requires substantial human resources and costs, which have limited their widespread adoption [6,7,8].

Delivering an intervention through unsupervised digital solutions (e.g., mobile applications) may offer a practical approach to overcoming these challenges. This approach reduces dependency on human supervision and resource-intensive management, addressing a key barrier to the broader implementation of multi-component interventions. Recent studies have explored the feasibility of digitalized multi-component interventions [9,10,11,12,13]. Okpara et al. offered a 12-week remote multi-component intervention including exercise coaching, medication counseling, and weekly check-ins [9]. Ohta et al. built a mobile app incorporating walking, cognitive training, and social communication for frail older adults [10]. Bajdek et al. supplied tablets and wearables that streamed video-guided exercises and weekly motivational calls [11]. Kwan et al. used an app to guide physical activity in cognitively frail older adults [12], and Li et al. combined a mobile app with wearable sensors to deliver physician-prescribed exercises [13]. These studies showed high adherence, typically above 80%, and modest gains in mobility or muscle strength. All, however, shared a key limitation that none of them were fully unsupervised. Each relied on some level of human contact, scheduled check-ins or extra hardware, factors that hinder large-scale rollout in community settings. To address this gap, the feasibility of a fully unsupervised intervention for older adults needs to be investigated. Therefore, in this study, we developed an app to deliver an unsupervised multi-component intervention for the prevention of frailty in older adults. We hypothesized that with appropriate design and support, older adults could safely use the app with high adherence, leading to improvements in physical performance. In addition, we explored whether the intervention could contribute to improving the status of frailty in terms of physical function, depressive symptoms, and self-efficacy. The primary objective was to evaluate the feasibility of this digital unsupervised multi-component exercise intervention in community-dwelling older adults by measuring the adherence rate of participants and changes from pre- and post-intervention in physical performance and other health outcomes.

## 2. Materials and Methods

### 2.1. Study Design

This study was designed as a single-arm pre–post interventional feasibility study. Participants underwent a 12-week unsupervised multi-component intervention using the app. Outcomes were assessed at pre- and post- 12 weeks intervention. The study was conducted in accordance with the Declaration of Helsinki and was approved by the Institutional Review Board of Kyung Hee University, Seoul, Republic of Korea (Approval No.: KHGIRB-22-326). Written informed consent was obtained from all participants prior to enrollment.

### 2.2. Participants

**Figure 1:**
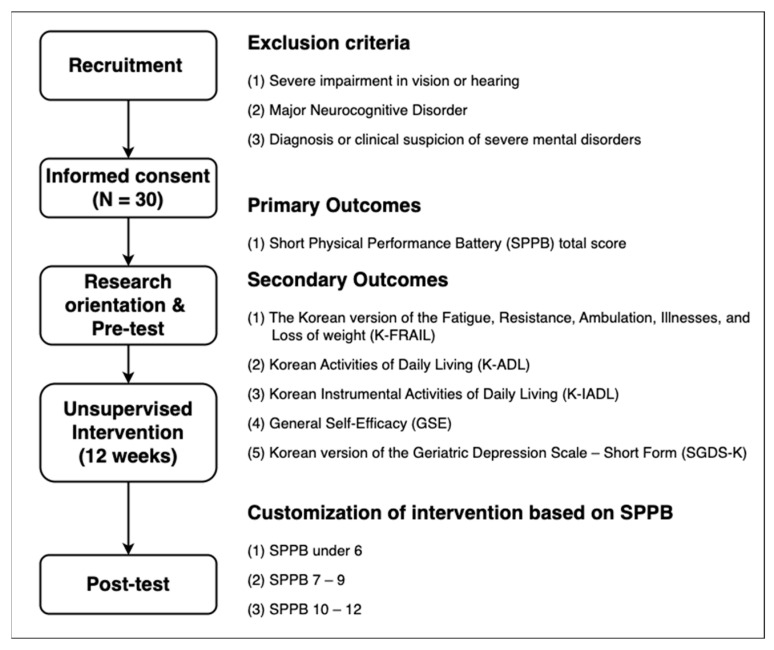
Study design for evaluating the feasibility of unsupervised multi-component intervention using a mobile application.

The participants were community-dwelling older adults aged 65 years or older. As shown in Figure 1, the participants were recruited from two social service centers in Seoul (Site A) and Incheon (Site B), in the Republic of Korea. Exclusion criteria were established to ensure the participants’ ability to operate the app and complete self-report measures independently. We therefore excluded (i) individuals with severe visual or hearing loss that would prevent them from following on-screen or audio instructions, (ii) those with major neurocognitive disorders that impair comprehension, and (iii) those with severe psychiatric disorders, to minimize safety risks even though the exercises were low-risk and unsupervised. A total of 39 older adults were screened for participation. Of these, 9 did not meet the inclusion criteria or declined to participate, and 30 eligible participants were enrolled in the study. All 30 participants completed the intervention and assessments. The mean age of the participants was 72.10 ± 4.54 years, and 25 participants (83.33%) were female. At pre-test, 11 participants (36.67%) had a Short Physical Performance Battery (SPPB) score of 7–9, indicating relatively low physical function, while 18 (60%) had an SPPB score of >9, indicating relatively higher physical function. Only 1 participant (3.33%) had an SPPB score of ≤6, which indicates severe frailty [1,2,3]. The characteristics of the participants are presented in Table 1.

### 2.3. Materials and Measures

#### 2.3.1. Mobile Application for Unsupervised Multi-Component Intervention

**Figure 2:**
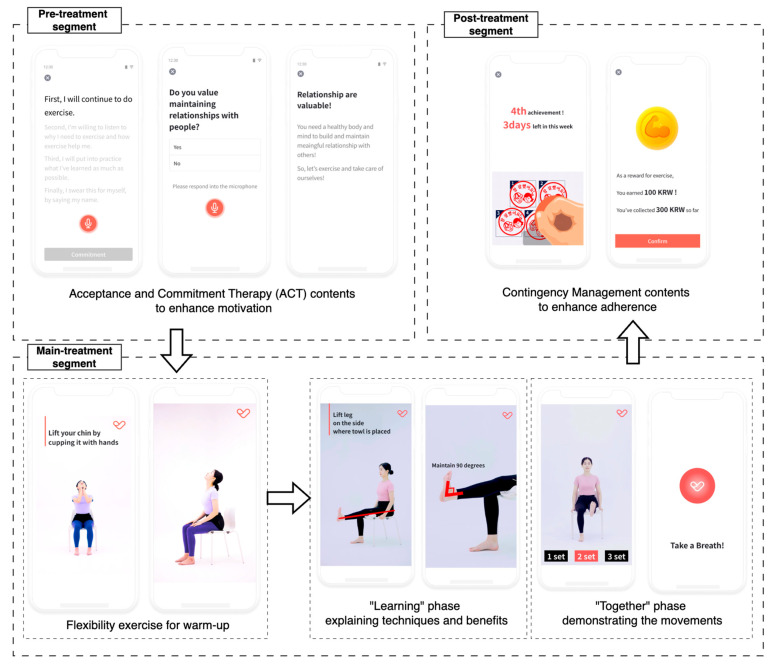
Structure of a session with pre-, main-, and post-treatment sections. The exercise contents in the main treatment section comprised two phases: “learning” and “together”.

We developed an app to deliver an unsupervised multi-component intervention for older adults [14]. The app provided daily coaching sessions that combined personalized exercise coaching based on SPPB scores with motivational and educational content to support adherence. Each daily coaching session was structured into three segments: (1) a pre-treatment segment for motivation, (2) a main treatment segment with guided physical exercises, and (3) a post-treatment segment for exercise feedback and reward (Figure 2). Participants were instructed to complete five sessions per week over 12 weeks, up to a total of 60 sessions.

The exercises in the main treatment segment were automatically selected according to each participant’s SPPB score, a standardized measure of physical function in older adults [1,15] (Table 2). Following established guidelines [1,2,15], participants were stratified into three groups based on their baseline SPPB scores (SPPB ≤ 6, 7–9 and 10–12) and each group received preset exercise contents matched to its functional level. To minimize the risk of falls or injury, most exercises were designed to require no special equipment. In particular, for participants with a lower SPPB score, the intervention emphasized seated or low-movement exercises to ensure safety. Additionally, the daily guidance was provided at the beginning of each main treatment segment, advising users to stop exercising immediately and contact the researcher or healthcare providers if they experienced severe pain or distress.

Each main treatment segment comprised two parts: (1) a flexibility warm-up and (2) a multi-component exercise block featuring three to four strength, balance, and aerobic exercises. The contents were delivered via instructional videos. Each video included a “learning” phase that explained benefits and correct technique, followed by a “together” phase in which a trainer demonstrated the movements while participants exercised simultaneously with real-time voice cues.

The app incorporated behavioral change strategies to facilitate adherence in the absence of direct human supervision. In particular, Acceptance and Commitment Therapy (ACT) [6] and Contingency Management (CM) [7] contents were presented during the pre- and post-treatment segments. The contents aimed to overcome psychological barriers to exercise by emphasizing personal benefits and values. After each completed main treatment segment, the app provided positive reinforcement in the form of verbal praise and a virtual “stamp” representing a virtual reward. If a participant accumulated five stamps within a 7-day period, they earned an additional virtual reward to further encourage continuous participation. The number of the additional virtual rewards increased if the participant consistently met the 5-session goal in subsequent weeks, incentivizing sustained adherence [8,16]. Over the 12-week intervention, a participant could earn up to 15,000 KRW (approximately 12 USD). At the end of the study, accumulated virtual rewards were exchanged for real gift certificates of equal value.

The user experience (UX) of the app was designed for older adults to minimize usability barriers related to their limited digital literacy [14,17]. Assuming the limited experience of older adults with smartphones or modern digital apps, the user interface (UI) elements, including button and page navigator, were simplified to ensure ease of use. Commonly used icons in apps, such as >(next), <(previous), ←(swipe), ≡ (menu), were omitted from the UI due to older adults’ unfamiliarity with these symbols [18]. Instead, text with clear instructions, such as “Start exercising today” or “See my info” were inserted into the UI to guide older adults on where to tap. In addition, buttons were highlighted in red to help older adults identify where to tap to proceed.

Furthermore, almost all user journeys in the app were designed to require only one or two taps. Some features allowed brief voice commands, such as “yes” or “no”, instead of tapping a button, recognizing that older adults are more familiar with verbal interactions and may have difficulty understanding the UI of app [19]. Important instructions were emphasized with text-to-speech audio and high-contrast color schemes. Interactions that may be unfamiliar or complicated for older adults, such as text typing, scrolling, and swiping, were excluded from all features of the app [18].

Throughout the 12-week period, participants were encouraged to use the app freely and complete as many daily coaching sessions as they could. The app automatically recorded every session completed for each participant. The intervention was designed with a target of 60 sessions over 12 weeks (5 sessions per week). Thus, adherence rate (%) was defined as:

(1) $$\mathrm{Adherence}\;\mathrm{rate}=\frac{Total\;number\;of\;sessions\;completed}{total\;number\;of\;sessions\;available\;to\;complete\;(60)}$$

The app contained a self-performed 30-second sit-to-stand (30STS) test module, completed in the home at baseline and at four-week intervals (i.e., week 0, 4, 8 and 12). After viewing an on-screen tutorial, participants sat on a chair, crossed their arms over their chests while holding the smartphone in their hands against the chest, and performed as many sit-to-stand repetitions as possible within 30 s. During each test, the phone’s triaxial accelerometer streamed data to an embedded peak-finding algorithm that automatically detect repetitions. If the algorithm failed to detect repetitions from the accelerometer signal, participants were prompted to repeat the test. However, in cases of repeated detection failure, the test was allowed to be skipped to prevent frustration or delays that might negatively impact adherence to the exercise intervention. This self-test module provided an objective, in-home measure of physical performance, allowing a continuous monitoring of progress throughout the intervention period.

#### 2.3.2. Short Physical Performance Battery (SPPB)

The SPPB includes 3 sub-tasks: (1) static balance, (2) 4 m gait speed, and (3) five times sit-to-stand, with a score ranging from 0 (worst) to 12 (best) [1]. Static balance is assessed by the ability to stand in three positions for 10 s each: (1) side-by-side, (2) semi-tandem, and (3) tandem. The 4 m gait speed is assessed by the time taken to walk 4 m at the participant’s usual pace. Five times sit-to-stand is assessed by the time required to rise from a chair and sit down five times as quickly as possible. In this study, the SPPB was assessed using a device for automated physical performance assessment (AndanteFit, DYPHI Inc., Daejeon, Republic of Korea) [3,20,21].

#### 2.3.3. Korean Version of the Fatigue, Resistance, Ambulation, Illnesses, and Loss of Weight (K-FRAIL)

The Korean version of the Fatigue, Resistance, Ambulation, Illnesses, and Loss of weight (K-FRAIL) questionnaire [22,23] was used to evaluate the self-reported frailty index of participants. The K-FRAIL consists of five items: (1) Fatigue: “How much of the time during the last month did you feel tired?” Responses of “all of the time” or “most of the time” were considered positive. (2) Resistance: “By yourself and without using aids, do you have any difficulty walking up 10 steps without resting?” A response of “yes” was considered positive. (3) Ambulation: “By yourself and without using aids, do you have any difficulty walking 300 m?” A response of “yes” was considered positive. (4) Illnesses: Presence of five or more of the following conditions: hypertension, diabetes, cancer (excluding minor skin cancer), chronic lung disease, heart attack, congestive heart failure, angina, asthma, arthritis, stroke, or kidney disease. (5) Loss of weight: Unintentional weight loss of more than 5% over the past year.

#### 2.3.4. Korean Activities of Daily Living (K-ADL) and Korean Instrumental Activities of Daily Living (K-IADL)

The Korean Activities of Daily Living (K-ADL) and Korean Instrumental Activities of Daily Living (K-IADL) [24] were used to measure the activities of daily living (ADL) and the instrumental activities of daily living (IADL) of participants. K-ADL comprised items checked for the independent functionality of the following daily behaviors: dressing, washing face and hands, tooth brushing, bathing, eating, transferring, toileting, stand up and sitting, move to sitting, leaving the room, toilet controlling, and washing hair. K-IADL comprised items checked for the independent functionality of the following instrumental daily behaviors: house working, preparing a meal, doing laundry, managing money, using a telephone, using public transportation, going out of the neighborhood, decorating, and taking medicines. All items of K-ADL and K-IADL were calculated on a scale from 0 to 2, with 0 indicating “totally independent”, 1 indicating “partially independent”, and 2 indicating “totally dependent”. The total score for the K-ADL scale was 0 to 26, while the K-IADL scale had a total score of 0 to 20.

#### 2.3.5. General Self-Efficacy (GSE)

The General Self-Efficacy scale (GSE) [25] was used to measure the self-efficacy of the participants. The GSE consists of 10 items, each ranging from 0 (strongly disagree) to 3 (strongly agree), that inquire about the ability to cope with a variety of challenging circumstances and to persist in achieving goals.

#### 2.3.6. Korean Version of the Geriatric Depression Scale-Short Form (SGDS-K)

The Korean version of the Geriatric Depression Scale-Short Form (SGDS-K) [26] was used to measure the depressive symptoms of participants. The SGDS-K consists of 15 items, each evaluated as either 1 (positive) or 0 (negative), with higher scores indicating more depressive symptoms. The optimal cutoff score representing clinically significant depression has been suggested to be 8 or higher.

### 2.4. Procedures

#### 2.4.1. Research Orientation and Pre-Test

All participants attended a group orientation at the start of the study. During the orientation, informed consent was obtained, and participants received an overview of the study procedures. Afterward, pre-test measurements were collected. Participants completed the K-FRAIL, K-ADL, K-IADL, GSE, and SGDS-K questionnaires with assistance from researchers as needed, after which the SPPB was administered under researcher supervision. Each participant’s SPPB score was then uploaded to the app server by the researchers to tailor the exercise program. Finally, to mitigate common difficulties older adults commonly face, such as finding an installed app and navigating unfamiliar features, as noted in a previous study [27], the researchers helped participants install the app on their smartphones and provided brief instruction in its use. Participants were also given a concise printed user guide for reference at home.

#### 2.4.2. Unsupervised Intervention for 12 Weeks

Participants were instructed to use the app independently for 12 weeks, aiming for 5 sessions per week. They were free to set their own schedules, as the study sought to observe natural usage and adherence in an unsupervised setting. No in-person supervision or coaching was provided during this period. We monitored app logs remotely to verify system performance and record usage, but we did not intervene on the basis of these data. Participants could contact the research staff by phone if they encountered technical difficulties with the app.

#### 2.4.3. Post-Test

Post-tests were conducted within seven days of completing the intervention. Post-tests were administered at the same social service centers where participants were originally recruited. The same outcome measures were administered: participants repeated the SPPB and completed the K-FRAIL, K-ADL, K-IADL, GSE, and SGDS-K questionnaires. After the post-test, each participant received a gift certificate equal to the virtual rewards accumulated in the app.

### 2.5. Statistical Analysis

All statistical analyses were performed in Python 3.13.1. To determine whether the outcome variables met the normality assumption, the Shapiro–Wilk test was conducted. The results indicated that none of the outcomes satisfied the normality assumption (*p* < 0.05). Therefore, the Wilcoxon signed-rank test was applied to each outcome to assess the effectiveness of the unsupervised intervention. Because only one participant fell into the SPPB under 7 group, the SPPB analyses were conducted after regrouping participants into two groups: SPPB ≤ 9 and SPPB > 9. Additionally, the Mann–Whitney U test was applied to compare the 30STS counts at week 0 between the groups.

## 3. Results

### 3.1. Adherence Rate

**Figure 3:**
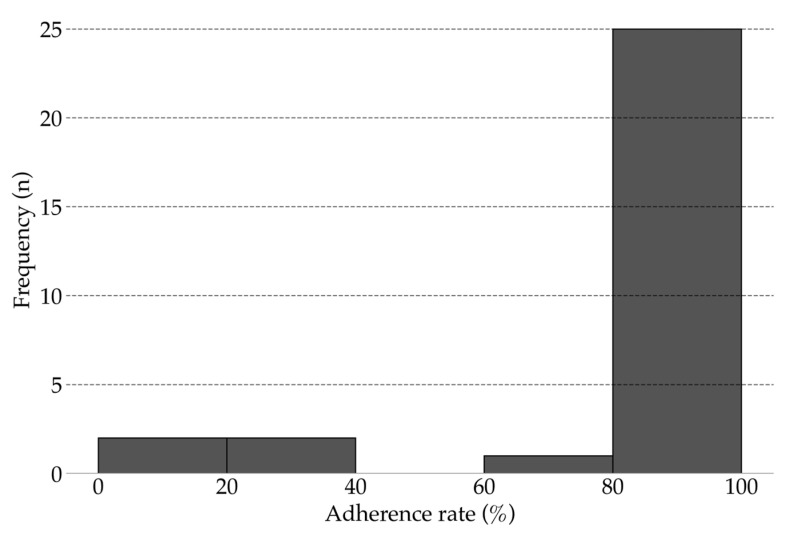
Adherence rate for unsupervised intervention (12 weeks).

All 30 participants completed 12-weeks of unsupervised intervention using the app. The mean adherence rate was 86.10% (range, 18.33–100%; 95% CI, 76.26–95.85; Figure 3). Overall, 83.33% of participants achieved an adherence rate above 80%. No injuries, falls, or other adverse events were reported during the study.

### 3.2. Self-Monitored Physical Performance

Table 3 summarizes the trajectory of 30STS test performance during the 12-week intervention. Because each test was self-performed at home, the number of samples at each time point fluctuated with participant availability. Baseline (week 0) 30STS counts did not differ significantly between the groups (*p* = 0.479). Thereafter, both groups demonstrated a progressive increase in 30STS counts, reflecting continual improvements in physical performance.

### 3.3. Primary Outcome

Table 4 summarizes the pre- and post-intervention SPPB results. Across all participants, the mean SPPB score increased significantly from 9.60 ± 1.62 to 11.73 ± 0.51 (mean Δ = 2.13; 95% CI 1.58–2.69). Participants with baseline scores ≤ 9 improved from 7.92 ± 1.26 to 11.58 ± 0.49 (mean Δ = 3.67; 95% CI 3.04–4.29), whereas those with scores > 9 improved from 10.72 ± 0.45 to 11.83 ± 0.50 (mean Δ = 1.11; 95% CI 0.82–1.40).

At the sub-task level, gait speed and sit-to-stand performance improved markedly. In the SPPB ≤ 9 group, gait speed scores increased from 2.83 ± 1.07 to 3.92 ± 0.28 (0.77 ± 0.23 m/s to 1.09 ± 0.13 m/s), and sit-to-stand scores improved from 1.75 ± 0.92 to 3.75 ± 0.43 (17.60 ± 4.46 s to 8.44 ± 2.76 s). In the SPPB > 9 group, gait speed scores increased from 3.50 ± 0.50 to 4.00 ± 0.00 (0.93 ± 0.25 m/s to 1.16 ± 0.20 m/s), and sit-to-stand scores improved from 3.33 ± 0.58 to 4.00 ± 0.00 (10.82 ± 2.18 s to 6.61 ± 1.12 s).

### 3.4. Secondary Outcome

Table 5 summarizes the secondary outcome measures of K-FRAIL, K-ADL, K-IADL, GSE, and SGDS-K. A significant reduction in geriatric depression was observed, with SGDS-K scores decreasing from 3.23 ± 3.08 to 2.00 ± 2.11 (mean Δ = −1.23; 95% CI −2.28–−0.18) after the intervention. No significant changes were detected in the other secondary outcomes.

## 4. Discussion

This study provides evidence that an unsupervised multi-component exercise intervention delivered via an app is both feasible and effective for preventing frailty in community-dwelling older adults. To our knowledge, this is one of the first studies to demonstrate that older adults can independently engage with a mobile-based remote intervention, achieving high adherence and improvements in physical function.

A key finding was the high adherence rate achieved without direct supervision. The average adherence rate was 86.10%, and 83.33% of participants achieved an adherence rate greater than 80%. Notably, this adherence was achieved without in-person coaching or frequent motivational contact. The adherence rate observed in this unsupervised intervention is comparable to rates reported in other digital exercise programs that included regular support from trainers or healthcare professionals [9,11,13].

As initial success in using digital services among older adults often leads to continued engagement [19], the high rate of adherence in this study reflects the successful initial adoption of the app. Several strategies implemented in the app may have contributed to this result. Firstly, by providing in-person installation and brief hands-on demonstrations during the orientation session, participants in this study faced no difficulty launching the app, and no participant failed to initiate its use even once. Secondly, the user interface and experience were tailored to accommodate the limited digital literacy of an older population. By eliminating complex icons and providing clear text and audio instructions, the app was made accessible to users with minimal technological experience. Thirdly, motivational and behavioral strategies were integrated into the app content. Messages based on ACT encouraged participants to reflect on personally meaningful reasons for exercising and managing discomfort [28,29]. Additionally, a CM-based reward schedule provided extrinsic motivation through points and feedback, promoting sustained engagement [11,30,31]. As a result, the high adherence observed in this unsupervised intervention highlights the importance of combining age-friendly design with evidence-based motivational and behavioral change strategies to effectively engage older adults in intervention without direct supervision.

Significant improvements in SPPB demonstrate the potential efficacy of this intervention in enhancing physical function. All participants, including those with relatively low baseline function (SPPB ≤ 9) [16,25], experienced significant gains (from 7.92 to 11.58) after 12 weeks. Improvements were particularly notable in gait speed and sit-to-stand performance. Importantly, even participants who were relatively robust at baseline (SPPB >9) also showed enhancements (from 10.72 to 11.83) in physical function. The average improvement of SPPB exceeded the threshold for clinically meaningful change, typically defined as an improvement of at least 1 point on the SPPB scale [30]. Notably, participants with low baseline SPPB scores improved to a degree comparable to that seen in traditional on-site interventions, where similar gains have been linked to meaningful health benefits such as the reversal of sarcopenia and reduced hospitalization rates [32,33,34]. These findings suggest that older adults can achieve clinically significant improvements—such as reduced risk of falls, lower mortality, and greater independence in daily activities [15,20,30]—even when the intervention is delivered without human supervision. The app’s capacity to automatically adjust exercise difficulty based on baseline ability may have contributed to these consistent outcomes across varying levels of initial function.

An implication of these findings is that mobile-based unsupervised interventions can yield outcomes comparable to traditional supervised programs [14,17]. Conventional multi-component interventions require substantial human resources, including trained personnel and frequent in-person visits, limiting their scalability [35,36,37]. In contrast, once developed, the app can be deployed widely at minimal marginal cost per additional user, independent of facility or instructor availability. These results indicate the feasibility of scaling up frailty interventions using technology. With further refinement and validation, this approach could be implemented in community and home settings to reach larger populations, particularly in areas with limited healthcare resources.

Although unsupervised multi-component intervention significantly improved physical function as measured by the SPPB, no statistically significant change was observed in the K-FRAIL. This discrepancy may reflect the inherent characteristics of the K-FRAIL as a screening tool rather than a responsive outcome measure [20,22]. The K-FRAIL includes items based on chronic conditions like comorbidities or recent unintentional weight loss which are less likely to change through short-term behavioral interventions. Furthermore, as a brief screening tool with binary responses, the K-FRAIL may lack responsiveness to detect small but meaningful improvements in function [38]. For instance, the fatigue item in the K-FRAIL asks whether the respondent has felt tired during the past month, rather than asking the degree in perceived fatigue. As a result, older adults who experienced increased physical engagement during the intervention may have still responded similarly at pre- and post-test, regardless of actual improvements in physical function. Moreover, K-FRAIL assesses physical function using two binary items—difficulty climbing 10 steps and walking 300 m—which may lack the sensitivity to reflect changes in physical function [39]. While the K-FRAIL may lack sensitivity to short-term functional improvements, its brevity and ease of use still make it valuable for initial frailty screening. Nonetheless, future studies aiming to detect more subtle changes may benefit from incorporating additional measures.

While participants reported low levels of depressive symptoms at pre-test, a small but statistically significant reduction in depressive symptoms (from 3.23 to 2.00) was still observed. Considering that the SGDS-K asks about the presence of specific depressive symptoms, this degree of improvement suggests that participants, on average, experienced at least one fewer clinically relevant depressive symptom. In terms of the diagnostic value of the SGDS-K, this improvement is comparable to a 6% reduction in the likelihood of being classified as having a major depressive disorder [40]. This finding aligns with prior evidence that physical activity and exercise benefit mood and psychological well-being [41,42]. The ACT-based content, designed to enhance psychological flexibility, may have contributed to this result by reducing negative emotions associated with exercise and aging [6,28]. However, given that very few participants reported clinically significant depressive symptoms (SGDS-K ≥ 8) at pre-test [26], the observed improvement–from low to even lower levels–may not indicate a clinically meaningful therapeutic effect. Future studies targeting older adults with greater psychological distress are warranted to clarify the mental health benefits of unsupervised multi-component interventions.

Despite improvements in physical function and depressive symptoms, there were no statistically significant changes in K-ADL, K-IADL, or GSE scores after the intervention. Examining the pre-test values, most participants already scored at the extreme ends in K-ADL (0.14 ± 0.58 out of 26), K-IADL (0.66 ± 2.64 out of 20), and GSE (28.61 ± 3.78 out of 30). Although definitive conclusions cannot be drawn, this suggests that the lack of statistically significant changes may be attributable to a ceiling effect. In other words, even if real improvements occurred, they may not have been captured due to the limited variability at baseline. These results may partly reflect the fact that the study recruited community-dwelling older adults who were able to actively engage with social service centers and voluntarily participate in research, suggesting that they were already able to manage daily tasks independently and had a strong sense of self-efficacy prior to the intervention. Therefore, future studies involving older adults with greater functional limitations or poor self-efficacy may offer further insight into the effects of unsupervised multi-component interventions.

This study integrated a self-performed physical performance test feature (i.e., 30STS test) to gather physical performance data under unsupervised conditions. By providing clear instruction and leveraging the triaxial accelerometer to capture participants’ movements with high precision, the app enabled the collection of meaningful physical performance data in the home environment without researcher supervision. These results indicate that the potentiality of mobile-based, unsupervised interventions extends beyond merely delivering exercise content, enabling an ongoing, personalized assessment of intervention effects throughout the program and suggesting a potential for a dynamic optimization of the intervention program in real time to each individual’s response. For instance, if a participant’s 30STS performance improves over time, the app could automatically adjust the intervention by increasing exercise difficulty (e.g., providing coaching with more repetitions, or more challenging exercises). This adaptive algorithm, which responds to performance data, highlights the considerable potential of unsupervised mobile-based intervention for scalable frailty management.

This study has several limitations that should be considered when interpreting the findings. Firstly, because it lacks a control group, it cannot establish strong causal inferences, and the observed changes may reflect unmeasured confounding factors. Secondly, although the SPPB improvement (mean Δ = 2.13, 95% CI 1.58 to 2.69) and adherence rate (mean = 86.05%, 95% CI 76.26 to 95.85) were estimated with reasonable precision, the small sample size still warrants caution. Thirdly, the sample was predominantly female (83.3%), which limits the applicability of the results to older men. Fourthly, recruiting through community centers yielded few frail individuals, few psychologically vulnerable participants, and few adults with severely low digital literacy. Consequently, the findings may not extend to these at-risk groups, and the effects on daily function and self-efficacy remain unclear. Fifthly, some participants experienced technical difficulties despite careful onboarding and a user-friendly interface. These problems included misunderstandings of the intervention structure, confusion while performing the 30STS test using only on-screen prompts, and occasional app errors, all of which led to the loss of some 30STS data. Sixthly, adherence was measured only through app-usage logs that recorded the completion of daily sessions, so the study could not verify whether participants actually performed the exercises. Objective monitoring methods, such as motion sensors or video recordings, were intentionally omitted to protect privacy and because many older adults have limited digital literacy. These issues highlight the need for future studies to find feasible ways to confirm exercise engagement objectively without compromising usability or privacy. Finally, more detailed analyses are needed to determine how much adherence-promoting components, such as ACT- and CM-based strategies, enhanced adherence beyond what might be achieved with exercise coaching alone.

## 5. Conclusions

This study demonstrates the feasibility and potential efficacy of an unsupervised multi-component exercise intervention delivered through the app for preventing frailty in community-dwelling older adults. The intervention resulted in a high adherence rate and clinically meaningful improvements in physical performance, even without direct supervision. These findings suggest that mobile-based unsupervised interventions can be a scalable and accessible strategy for frailty prevention and management in older populations.

## Figures and Tables

**Table 1:** Characteristics of participants.

Characteristics	Values
Age (year)	72.10 ± 4.50
Sites and Sex	
Site A	11 (36.70%)
Male	1 (9.10%)
Female	10 (90.90%)
Site B	19 (63.30%)
Male	4 (21.10%)
Female	15 (78.90%)
Physical function	
SPPB under 6	1 (3.33%)
SPPB 7–9	11 (36.67%)
SPPB 9–12	18 (60.00%)

SPPB—Short Physical Performance Battery.

**Table 2:** List of multi-component exercises designed in this study tailored to functional level.

SPPB	Resistance	Aerobic	Balance	Flexibility
Under 7	Glute bridge, Seated leg extensions Bicep curls, Squeeze a ball, Seated heel raise,	Seated march	Lateral Walk, Single Leg Standing	Chest/upper back stretch, Waist/ribcage stretch Hip stretch, Quad stretch, Hamstring stretch, Calf stretch, Neck stretch, Shoulder stretch, Wrist stretch
7–9	Sit to stand, Standing side leg raise, Standing heel raise, Twist a towel, Overhead shrug,	Standing march	8-line walking, Single leg standing with high knee	
10–12	Wall squat, Wall push up, Seated knee up, Standing backward leg raise, Assisted chair lunge, Bent over triceps extension	Standing march with high knee	Heel-to-toe walk	

SPPB—Short Physical Performance Battery.

**Table 3:** Progression of 30 s sit-to-stand counts over the intervention period (12 weeks).

Groups	30 S Sit-to-Stand Counts							
	Week 0		Week 4		Week 8		Week 12	
	*n*	M ± SD	*n*	M ± SD	*n*	M ± SD	*n*	M ± SD
SPPB ≤ 9	8	11.63 ± 2.77	4	13.00 ± 2.16	1	21.00	0	-
SPPB > 9	11	13.36 ± 4.80	4	13.50 ± 4.51	6	18.33 ± 10.35	1	11.00

SPPB—Short Physical Performance Battery.

**Table 4:** Changes in SPPB scores before and after the 12-week unsupervised intervention.

	Entire (*n* = 30)				SPPB ≤ 9 (*n* = 12)				SPPB > 9 (*n* = 18)			
	Pre	Post	Mean Difference	Z	Pre	Post	Mean Difference	Z	Pre	Post	Mean Difference	Z
SPPB score	9.60 ± 1.62	11.73 ± 0.51	2.13 ***	−4.67	7.92 ± 1.26	11.58 ± 0.49	3.66 **	−3.13	10.72 ± 0.45	11.83 ± 0.50	1.11 ***	−3.70
Static balance score	3.67 ± 0.70	3.87 ± 0.43	0.2	−1.22	3.33 ± 0.94	3.92 ± 0.28	0.066	−1.84	3.89 ± 0.31	3.83 ± 0.50	−0.06	−0.38
Side-by-side (s)	10.00 ± 0.00	10.00 ± 0.00	−	−	10.00 ± 0.00	10.00 ± 0.00	−	−	10.00 ± 0.00	10.00 ± 0.00	−	−
Semi-tandem (s)	9.97 ± 0.18	10.00 ± 0.00	0.03	−1.00	9.92 ± 0.27	10.00 ± 0.00	0.08	−1.00	10.00 ± 0.00	10.00 ± 0.00	−	−
Tandem (s)	8.97 ± 2.48	9.38 ± 1.94	0.41	−0.89	7.78 ± 3.46	9.59 ± 1.36	1.81	−1.76	9.77 ± 0.84	9.24 ± 2.23	−0.53	−0.73
Gait speed score	3.23 ± 0.84	3.97 ± 0.18	0.74 ***	−3.82	2.83 ± 1.07	3.92 ± 0.28	1.09 *	−2.57	3.50 ± 0.50	4.00 ± 0.00	0.50 **	−3.00
4 m gait speed (m/s)	0.87 ± 0.25	1.13 ± 0.18	0.026 ***	−4.34	0.77 ± 0.23	1.09 ± 0.13	0.32 **	−3.06	0.93 ± 0.25	1.16 ± 0.20	0.23 **	−3.10
Sit-to-stand score	2.70 ± 1.07	3.90 ± 0.30	1.20 ***	−4.19	1.75 ± 0.92	3.75 ± 0.43	2.00 **	−3.03	3.33 ± 0.58	4.00 ± 0.00	0.67 **	−3.21
5x sit-to-stand (s)	13.53 ± 4.67	7.34 ± 2.14	−6.19 ***	−4.78	17.60 ± 4.46	8.44 ± 2.76	−9.16 **	−3.06	10.82 ± 2.18	6.61 ± 1.12	−4.21 ***	−3.72

SPPB—Short Physical Performance Battery; * *p* < 0.05 ** *p* < 0.01 *** *p* < 0.001.

**Table 5:** Changes in secondary outcomes before and after the unsupervised intervention.

	Pre	Post	Mean Difference	Z
SGDS-K	3.23 ± 3.08	2.00 ± 2.11	−1.23 *	−2.30
K-FRAIL scale	0.73 ± 0.77	0.47 ± 0.72	−0.26	−1.66
K-ADL	0.14 ± 0.58	0.10 ± 0.56	−0.04	−1.00
K-IADL	0.66 ± 2.64	0.69 ± 2.17	0.03	−0.09
GSE	28.61 ± 3.78	29.14 ± 5.99	0.53	−0.63

SGDS-K—The Korean Version of the Geriatric Depression Scale-Short Form; K-FRAIL—Korean Version of the Fatigue, Resistance, Ambulation, Illness, and Loss of weight; K-ADL—Korean Activities of Daily Living; K-IADL—Korean Instrumental Activities of Daily Living; GSE—General Self-Efficacy; * *p* < 0.05.

## Data Availability

The datasets used and analyzed during the current study are available from Changseok Lee on reasonable request.
